# The RNA interactome in the Hallmarks of Cancer

**DOI:** 10.1002/wrna.1786

**Published:** 2023-04-12

**Authors:** Marta M. Gabryelska, Simon J. Conn

**Affiliations:** ^1^ Flinders Health and Medical Research Institute (FHMRI) College of Medicine and Public Health, Flinders University Bedford Park South Australia Australia

**Keywords:** Hallmarks of Cancer, molecular biology, RNA interactomics, RNA–protein, RNA–RNA

## Abstract

Ribonucleic acid (RNA) molecules are indispensable for cellular homeostasis in healthy and malignant cells. However, the functions of RNA extend well beyond that of a protein‐coding template. Rather, both coding and non‐coding RNA molecules function through critical interactions with a plethora of cellular molecules, including other RNAs, DNA, and proteins. Deconvoluting this RNA interactome, including the interacting partners, the nature of the interaction, and dynamic changes of these interactions in malignancies has yielded fundamental advances in knowledge and are emerging as a novel therapeutic strategy in cancer. Here, we present an RNA‐centric review of recent advances in the field of RNA–RNA, RNA–protein, and RNA–DNA interactomic network analysis and their impact across the Hallmarks of Cancer.

This article is categorized under:

RNA in Disease and Development > RNA in Disease
RNA Interactions with Proteins and Other Molecules > RNA–Protein Complexes

## Graphical abstract

Interactions of RNA molecules play key roles across all Hallmarks of Cancer and were not captured by large genome sequencing efforts. These RNA interactomics provide novel insight into cancer initiation and progression, and provide novel therapeutic targets for treatment of cancers and many other diseases.

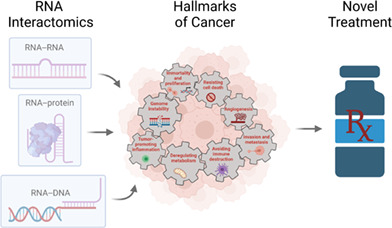

## 1 INTRODUCTION

Without question, the outcomes from the Human Genome Project have altered the landscape of clinical research. However, it did not meet the lofty aims espoused by many scientists and politicians, including President Bill Clinton, of uncovering the basis of most, if not all, hereditary diseases. Ten years after its completion, one review of this pioneering project in the journal *Nature* (Manolio et al., 2009), summarized that while over $100 billion was spent on this endeavor and despite >700 genome‐scanning publications emerging from this study to date, geneticists had liberated only a fractional genetic basis for human disease. What was critical about this review was one of the authors, Francis Collins, was one of the few remaining scientifically active leaders of the original Human Genome Project. For many, the assumption from the inability to identify genes for common diseases from these projects is that they do not exist, leading to the re‐prioritization of antireductionist science, including systems biology, to explain these conditions and seek additional therapeutic targets.

**FIGURE 1:**
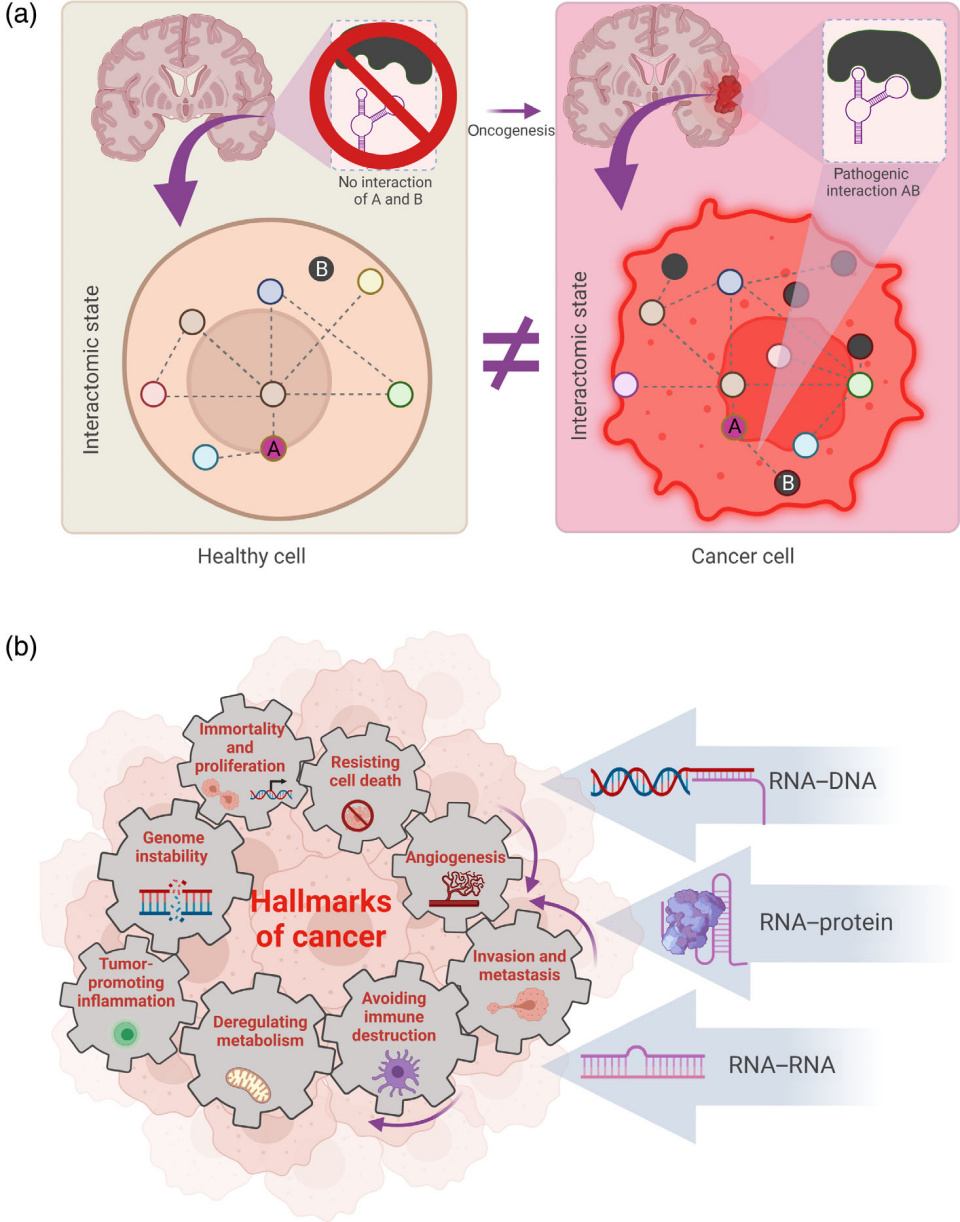
RNA interactions and their involvement in cancer. (a) Comparison of healthy (brown) and cancerous (red) cell interactomes in a hypothetical tumor. During oncogenesis, the RNA interactome (dotted lines) shifts with the changes of gene expression (gene product showed as a circle referring to any RNA, DNA, or protein molecule). Disrupting or preventing pathogenic interactions can become a future therapeutic strategy. (b) RNA–DNA, RNA–protein, and RNA–RNA interactions within the interdependent hallmarks of cancer. Created with BioRender.com.

The advent of high‐throughput RNA sequencing and quantitative proteomics were exploited to profile tens‐of‐thousands of individuals, to complement the Human Genome Project by illuminating the importance of epigenomic, transcriptomic, and proteomic expression profiling in development and disease. As expected, this broader profiling, paired with improved bioinformatic analysis, has valorized projects like The Cancer Genome Atlas with improvements in the ability to diagnose, treat, and prevent cancers. Yet, these studies do not investigate the static or dynamic interactions between these “omes.” With the lifetime risk of developing cancer currently at one in every two people and the lifetime risk of dying from cancer at one in five people (Ahmad et al., 2015), further illumination of interactomes at the highest achievable resolution is not only likely to achieve step‐changes in cancer treatments but also is necessary to revolutionize our understanding of and therapeutic potential for all forms of disease (Figure 1a).

Hallmarks of cancer are 10 ingrained, cellular defense mechanisms that are breached during oncogenesis, including: (1) sustained proliferative signaling, (2) evading growth suppressors, (3) enabling replicative immortality, (4) avoiding immune destruction, (5) genome instability and mutations, (6) tumor‐promoting inflammation, (7) activating invasion and metastasis, (8) inducing angiogenesis, (9) resisting cell death, and (10) deregulating cellular energetics (Hanahan & Weinberg, 2000, 2011; Figure 1b). The collective understanding of the structure and nature of protein interaction networks is among the best appreciated of biological networks, particularly given the rich data set of protein interactions that are available in a range of cell types and disease states (Taylor & Wrana, 2012) up to a single‐cell level (Mohammadi et al., 2019). It was elegantly demonstrated that genetic alterations in the most extensively rewired protein–protein interaction network nodes occur frequently in colorectal cancer, esophageal adenocarcinoma, and are predictive for poor patient outcomes (Al‐Harazi et al., 2021; Kennedy et al., 2020; Lv et al., 2019; Rezaei‐Tavirani et al., 2017; Yue et al., 2020). Comprehensive multiomics data obtained through the sequencing of tumor samples and experimental model systems will be important in implementing novel cancer systems biology approaches and increasing their efficacy for tailoring novel personalized treatment modalities in cancer (Yalcin et al., 2020). A diversity of methods in cancer systems biology has been described which use large datasets to elucidate the molecular networks by which cancer develops (Kuenzi & Ideker, 2020; Suhail et al., 2019). So called “functional variomics” is crucial to understanding the complex pleiotropic effect of cancer genes and provides a possible link between genotype and phenotype in cancer (S. Yi et al., 2017). However, none of the approaches mentioned above currently incorporate RNA interactomics.

**FIGURE 2:**
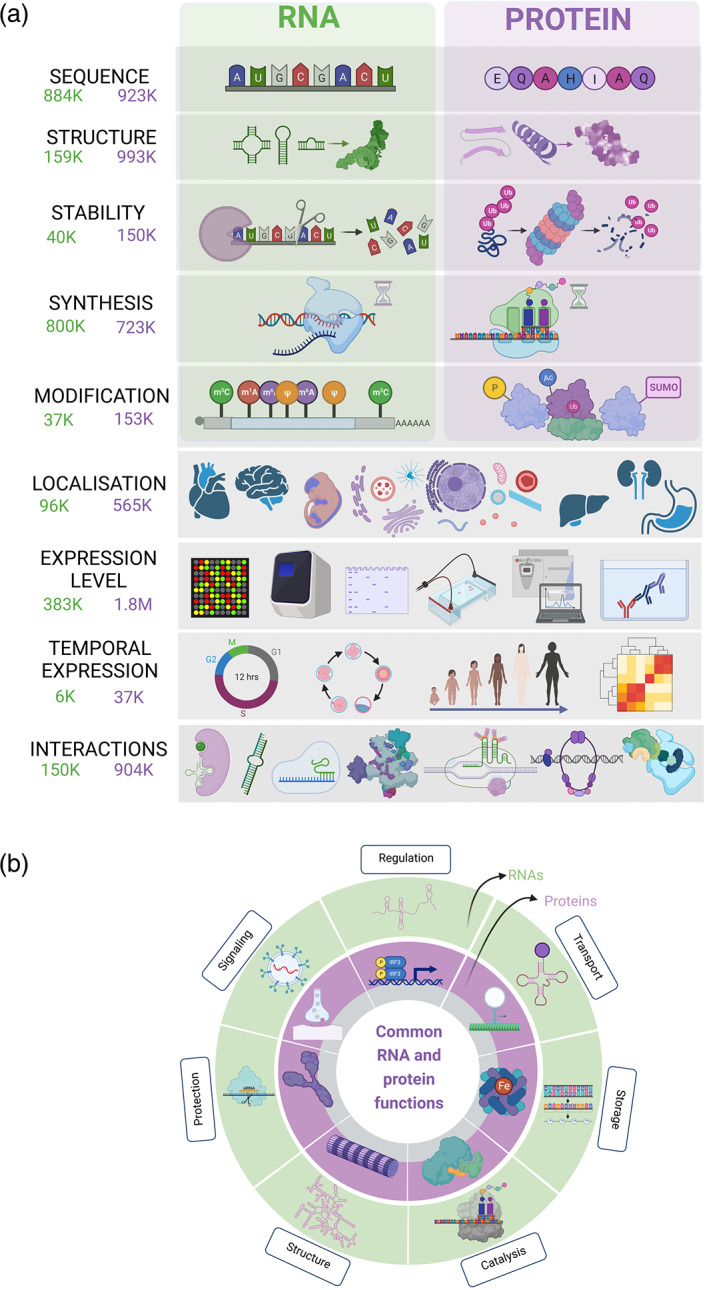
Comparison of properties and functions characterizing RNA and protein molecules. (a) Common descriptors of RNA and proteins sequence, structure, stability, synthesis, modification, localization, level, timing, and interactions. RNA‐ and protein‐specific properties with green and purple background (sequence, structure, stability, synthesis, modification). All of the presented properties are not adequately explored for RNA molecules. NCBI PubMed database was searched using the terms “cancer” + [“RNA” or “protein”] + “feature.” The corresponding number of publications covering each topic in relation to cancer studies is presented on the left (RNA in green, protein in purple). (b) Common functions of RNA (outer light‐gray circle) and proteins (inner dark‐gray circle) with illustrated examples. Created with BioRender.com.

While scientific attention has been focused on the roles of proteins in the hallmarks of cancer (1.45 M publications on PubMed, November 2021), there is growing awareness of the importance of RNA molecules (281 k publications). RNA and proteins are usually described as a messenger of genetic information (RNA) and cellular building blocks (proteins). The roles of proteins in cancer are commonly assessed at the level of abundance, structure, function, substrate binding, interaction profile, complex formation, and location (Figure 2a). Furthermore, despite possessing similar functions as proteins (Figure 2b), RNA molecules remain inadequately explored. This gap in knowledge necessitates investigation as it offers a vast source of potentially novel therapeutic targets in cancer. RNA has a great capacity to rapidly and dynamically change in response to environmental changes—including by changing confirmation, nucleotide modifications, overcoming the limitations imposed by being encoded by DNA. Many examples of RNA as a master molecular regulator, including ribozymes (Cech & Bass, 1986), RNA interference (Fire et al., 1998), riboswitches (Winkler et al., 2002), and clustered regularly interspaced short palindromic repeats (CRISPR; Jinek et al., 2012) have been illuminated in the past decade.

Any change in the gene expression level affects the delicate balance of interactions in the cells. Exploiting these interactions can allow the identification of functional target sites for future gene therapy and the verification of suspected RNA functions in cancer. Nevertheless, research focused on molecular interactions is scarce due to the high cost and technical and analytical challenges. Therefore, we cannot currently fully comprehend the role of dynamic RNA networks in mapping genotypes to phenotypes.

## 2 SIGNIFICANCE OF THE RNA INTERACTOME FOR CANCER STUDIES

The structure of RNA was previously shown to be critical for its functional interactions with other RNAs and protein targets within the cell, including functional folding of catalytic RNAs (e.g., ribozymes; Gabryelska et al., 2013), interactions of viral RNAs with host cellular components (Ziv et al., 2018) and the ability of microRNAs to bind the 3′ untranslated region of messenger RNA (Abulwerdi et al., 2019; Z. Lu et al., 2016; Mustoe et al., 2018; Roy et al., 2019). Interactomic studies have shown great promise in slowly deconvoluting the roles of molecular networks in fundamental biological processes (Figure 2b). Still, how interactomes differ between tumor and “normal” tissue, or how oncogenic mutations impact these interactions and their functions at a network‐level scale, is poorly understood.

Interactions between RNA molecules themselves, with proteins, DNA, and small molecules control the flow of molecular information within cells. Human DNA encodes around 20,000 protein‐coding genes. Considering products of alternative splicing, those containing single amino acid polymorphisms arising from nonsynonymous single‐nucleotide polymorphisms, and those that undergo post‐translational modifications, it has been estimated that as many as 100 different proteins can potentially be produced from a single gene (Ponomarenko et al., 2016), resulting in >2 million distinct hypothetical proteins. It was calculated that approximately as much as 77% of the human genome is transcribed and that protein‐coding genes contribute to only 5% of genome‐transcribed bases (detected uniquely in polyA‐prepared libraries; G. Liu et al., 2013). For 20,000 human protein‐coding genes there are almost 150,000 transcript isoforms (W. Jiang & Chen, 2021) and over 30% of tissue‐dependent transcript variations constituted by local splicing variations (Vaquero‐Garcia et al., 2016). Additionally, the CHESS project, combining sequencing data from thousands of samples from genotype‐tissue expression (GTEx; The GTEx Consortium et al., 2015) study (~900 billion reads) detected around 350,000 annotated transcripts and over 30 million additional transcripts at more than 650,000 genomic loci (Pertea et al., 2018). This makes an accurate estimation of possible functional transcripts and the level of transcriptional noise, increasingly difficult, putting in perspective what we already know about transcriptional complexity.

The function of all these molecules is based on their interactomic potential—which is embedded in their structure, flexible conformations, availability, accessibility, and spatiotemporality. Allowing unique combinations of just two RNA molecules (out of only 1 million), there are half a trillion possible combinations (5 × 10^11^), but this increases to 170 quadrillion (1.7 × 10^17^) possibilities where any three RNA transcripts are considered. These estimates are overly conservative, as they do not capture the potential interaction sites within the individual long RNA transcripts, multiple interacting partners, strength, specificity, saturation, and duration of interaction. However, it provides a sense of the scale of functional information which remains to be illuminated and obviates a need for artificial intelligence (AI) into ‐omics and, specifically interactomics, studies (discussed later).

There is a great need to understand the fundamentals of cancer and its progression to currently incurable states to develop new and more effective targeted therapies. Therefore, it is critical to expand our knowledge of molecular functions in pathogenesis and progression of cancer through the lenses of RNA systems biology. The purpose of this review is to highlight how the RNA interactome can be profiled and the role of specific RNA interactomes in the hallmarks of cancer.

## 3 METHODOLOGICAL ADVANCES IN RNA INTERACTOME CAPTURE

### 3.1 Capturing RNA–RNA interactions

**FIGURE 3:**
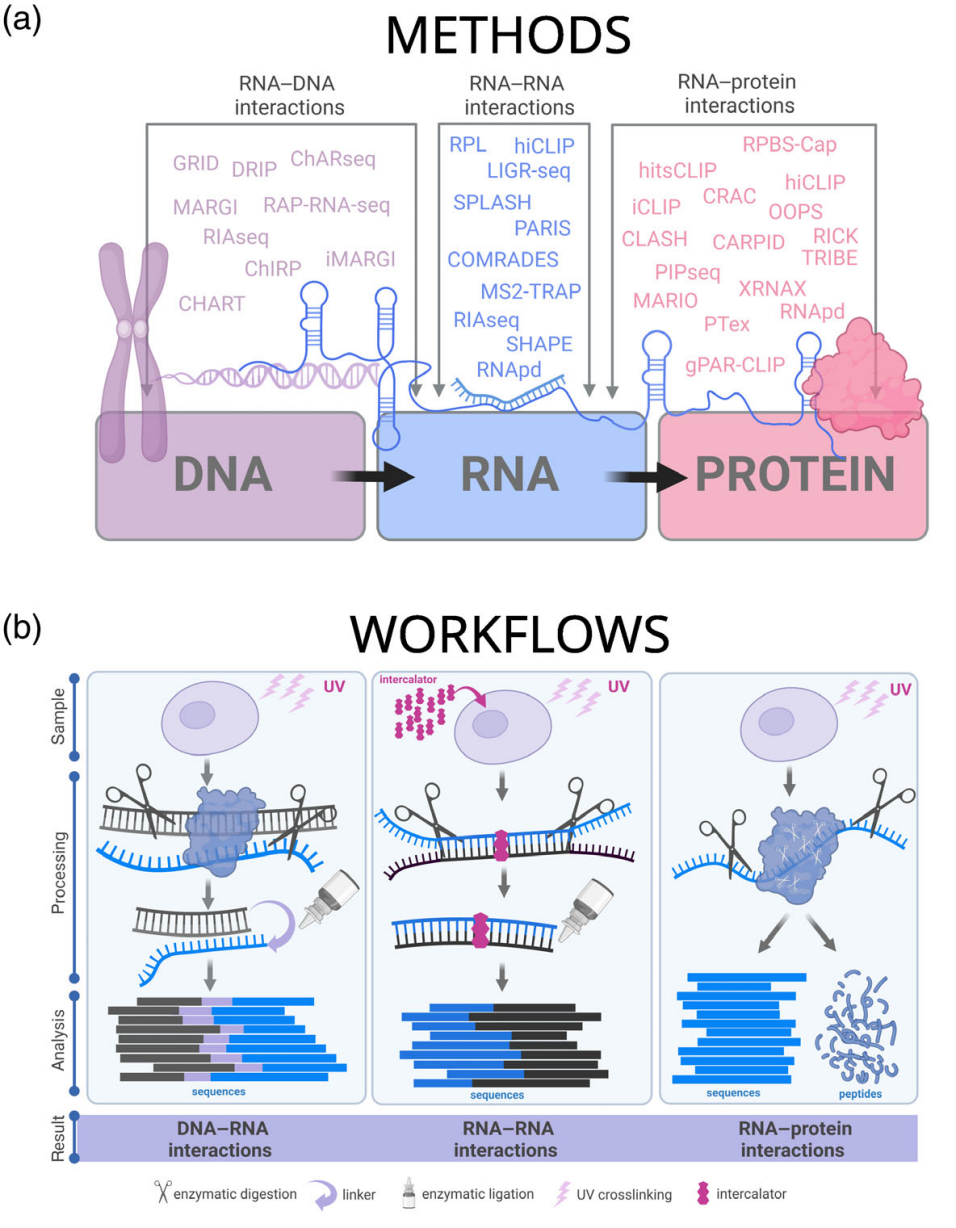
Methodological approaches to study RNA interactions. (a) Methods used to study RNA–centric interactions between DNA, RNA, and protein molecules with the arrows highlighting the interacting partners. (b) General strategies in high‐throughput methods for tracing RNA–DNA (left), RNA–RNA (middle), and RNA–protein (right) interactions. Many techniques employ ultraviolet light (UV light, purple) to covalently capture interactions. The subsequent step involves isolation of interacting material and trimming (scissors), often followed by joining (ligating, depicted with glue bottle and purple arrow) interacting partners. Nucleic acid interactions are analyzed via next‐generation sequencing (reads presented as rectangular blocks), while protein interactomes might involve mass spectroscopy of peptides to identify interacting proteins. DRIP, DNA–RNA immunoprecipitation; MARGI, mapping RNA–genome interactions; GRID, global *RNA* interaction with *DNA* sequencing; iMARGI, in situ mapping RNA–genome interactions; ChARseq, chromatin‐associated RNA sequencing; CLASH, cross‐linking, ligation, and sequencing of hybrids; CRAC, in vivo crosslinking and cDNA analysis; iCLIP, individual‐nucleotide resolution cross‐linking and immunoprecipitation; hitsCLIP, high‐throughput sequencing of RNA isolated by crosslinking immunoprecipitation; hiCLIP, hybrid and individual nucleotide resolution ultraviolet crosslinking and immunoprecipitation; OOPS, orthogonal organic phase separation; PTex, Phenol Toluol extraction; CARPID, CRISPR‐assisted *RNA*–*protein* interaction detection; RNApd, RNA pull‐down; TRIBE, targets of RNA‐binding proteins identified by editing; global mapping of RBP–RNA interactions, including global PAR‐CLIP (gPAR‐CLIP); protein interaction profile sequencing (PIP‐seq); mapping RNA interactome in vivo (MARIO); RNA using click chemistry (RICK); capture of protein‐binding sites on RNAs assay (RPBS‐Cap); capture hybridization analysis of RNA targets (CHART); chromatin isolation by RNA purification (ChIRP); RNA antisense purification sequencers (RAP RNA‐seq); RNA interactome analysis with new generation sequencers (RIA‐seq); eCLIP, enhanced CLIP; irCLIP, infrared CLIP; sCLIP, simplified CLIP; MS2‐TRAP, MS‐tagged RNA affinity purification. Created with BioRender.com.

One of the characteristic features of RNA is its flexibility, making its structure dynamically responsive and strongly affected by the cellular environment (Beaudoin et al., 2018; Guo & Bartel, 2016; Mizrahi et al., 2018; Spitale et al., 2015). RNA molecules drive robust cellular processes and most of their functions require interactions with other RNA molecules. There are many genome‐wide approaches to map these cellular RNA–RNA interactions (Aw et al., 2016; Kudla et al., 2011; Z. Lu et al., 2016; Ramani et al., 2015; Sharma et al., 2016; Ziv et al., 2018; Figure 3a). These studies enrich our fundamental knowledge of the impact of the cellular environment on RNA structure and explore how changes in RNA structure mediate alternative functions. Most of the recent RNA–RNA interactomic studies use psoralen, a hydrophobic chemical compound that intercalates into double‐stranded nucleic acids. Upon photoactivation with long‐wavelength UV light (365 nm), psoralen crosslinks opposite strands of RNA or DNA in a two‐step reaction (Figure 3b; Z. Lu et al., 2016). Crosslinked RNA strands are covalently frozen at the moment of interaction and unlike in the case of other chemical and enzymatic methods to probe RNA structure (with the use of dimethyl sulfate, lead ions, free radicals, ribonucleases; Deigan et al., 2009; S. E. Wells et al., 2000; Ziehler & Engelke, 2000), RNA proximity ligation methods illuminates direct interacting partners. Critically, following fragmentation of the RNA and ligation of interacting partners, the psoralen cross‐link is photo reversible with the use of shorter‐wavelength ultraviolet light, necessary to permit reverse transcription and high‐throughput sequencing to identify these interactions.

### 3.2 Capturing RNA–protein interactions

RNA–protein interactions are mediated by transient non‐covalent interactions such as electrostatic interactions and hydrogen bonds between specific residues in RNA and protein molecules. RNA binding proteins (RBPs) are necessary for a range of cellular functions—including cell transport, localization, development, differentiation, metabolism, post‐transcriptional regulation, formation and function of transcripts, cell homeostasis, RNA splicing, polyadenylation, mRNA stability, mRNA localization, and translation through interacting with coding and non‐coding RNAs, and other proteins (D. Kang et al., 2020; Qin et al., 2020). Not only are these processes perturbed in cancer cells, RBPs themselves show higher rates of mutation than transcription factors in breast, bladder, cervical, and brain cancer (Neelamraju et al., 2018), potentially affecting the scope of available interactions.

Shortwave UV radiation can induce covalent bond formation between two closely placed aromatic rings. Aromatic ring structures are found in several amino acids in proteins and nitrogenous bases in nucleic acids. Therefore, UV irradiation is used to covalently link RNA and interacting proteins, whereby the RNA–protein complexes can be further isolated and analyzed. A seminal study by Castello et al. (2012) utilized two complementary approaches for UV crosslinking of RBPs to RNA which greatly expanded the repertoire of human RBPs using the human cervical adenocarcinoma cell line, HeLa cells. Proximity ligation, in which physically close RNA fragments are ligated to one another, has been initially used with a protein decoy to detect long‐range interactions (Imig et al., 2015; Kudla et al., 2011; Sugimoto et al., 2015; Figure 3). RNA–protein crosslinking exists in the most commonly used protocols like CLIP (cross‐linking immunoprecipitation) (Imig et al., 2015), CRAC (cross‐linking and cDNA analysis) (Ule et al., 2003), and CLASH (cross‐linking, ligation and sequencing of hybrids) (Kudla et al., 2011). Development of the XRNAX (protein‐crosslinked RNA extraction) method, which employs an elegant modification of the TRIzol extraction method to enrich for RNA–protein complexes has been shown to illuminate the bound proteome at greater depth (Trendel et al., 2019; Xiao et al., 2019).

Recently, a family of new, improved protocols to capture RNA–protein interactions was reviewed (C. Lin & Miles, 2019). Orthogonal organic phase separation (OOPS) does not require molecular tagging or capture of polyadenylated RNA (Queiroz et al., 2019). It recovers cross‐linked protein–RNA and free protein, or protein‐bound RNA and free RNA, in an unbiased way and can enable analyses of dynamic RNA–protein interactions. UV‐dose‐dependent migration of RNA from the aqueous phase to the interface, saturating at ~75% of the total RNA content emphasized the load of RNA–protein interactions in living cells. The phenol toluol extraction (PTex) protocol does not rely on a specific RNA sequence or motif for isolation of cross‐linked ribonucleoproteins (RNPs), but rather purifies them based entirely on their physicochemical properties (Urdaneta et al., 2019).

Recently developed CRISPR‐assisted RNA–protein interaction detection method (CARPID), leverages CRISPR–CasRx‐based RNA targeting and proximity labeling to identify binding proteins of specific long non‐coding RNAs (lncRNAs) in the native cellular context (W. Yi et al., 2020). The authors found and verified that TAF15 (TATA‐box Binding Protein Associated Factor 15) and SNF2L (SWI/SNF‐related Matrix‐associated Actin‐dependent Regulator of Chromatin) bind *XIST* lncRNA, supporting a model in which both recruiting repressive factors (e.g., SNF2L) and evicting transcription activators (e.g., TAF15) confer *XIST*‐mediated XCI, consistent with previous models (Jégu et al., 2019; Minajigi et al., 2015).

Evaluation of these RNA–protein capture methods show that there is a need for simultaneous identification and analysis of RNA and protein partners to report the exact RNA and amino acid sequence involved in the interaction.

### 3.3 Capturing RNA–DNA interactions

The concept of chromatin‐associated (caRNA) or chromosomal RNA (cRNA) was introduced in 1970, but for many years the interaction was elusive (Nozawa & Gilbert, 2014). RNA has a great potential to interact with DNA as it can easily base pair with a reverse‐complementary fragment of melted, single‐stranded DNA or double‐stranded DNA, forming an RNA–DNA triplex. These interactions, commonly involving proteins, ultimately have the potential to affect gene expression. In a pathological context, perturbation or mutation of any of the following factors causes the chromosomal accumulation of RNA–DNA hybrids and consequent genomic instability: (1) mRNA splicing factors and RNA export factors, (2) RNA–DNA hybrid helicases, (3) RNA–DNA ribonucleases, (4) homologous recombination proteins, (5) Fanconi anemia proteins, and (6) topoisomerases (Halász et al., 2017).

Within the last 15 years, a series of methods have been developed to capture and understand RNA‐chromatin interactions (Figure 3a). The DRIP (DNA–RNA immunoprecipitation) family of methods utilize the S9.6 anti‐RNA–DNA hybrid antibody (Z. Hu, 2006) to capture RNA–DNA hybrids in their native chromosomal context. It does not offer explicit capture of directly interacting partners, but points at particular genomic regions enriched in deep sequencing (Halász et al., 2017). MARGI (Mapping RNA–genome interactions; Sridhar et al., 2017) and ChARseq (chromatin‐associated RNA sequencing; Bell et al., 2018) were the first methods to capture specific RNA–DNA interactions. The enhanced version iMARGI (in situ MARGI; W. Wu, Yan, et al., 2019; Z. Yan et al., 2019) proved that 50% of the most significant RNA–DNA interactions in normal cells colocalized with the gene pairs that form fusion RNAs in cancer. It suggested that spatial proximity of RNA and DNA could poise for the creation of fusion transcripts. GRID (global RNA interactions with DNA by deep sequencing) provides a strategy for de novo identification of RNA–DNA interactions (Zhou et al., 2019). The methodology assumes no direct interaction between the strands, but rather their proximity based on common protein interaction. It is based on the use of biotinylated bivalent linkers that are ligated to both RNA and DNA in situ, subsequently allowing affinity purification and distinguishing between the strands in sequencing data, without requiring any information on the bridging protein.

These methods allow us to study the complex processes of gene regulation in the context of chromatin architecture. However, the limitations are related to the initial cellular input and amount of RNA molecules involved in the interaction.

## 4 RNA INTERACTIONS IN THE HALLMARKS OF CANCER

### 4.1 Multi‐influential interactions

**FIGURE 4:**
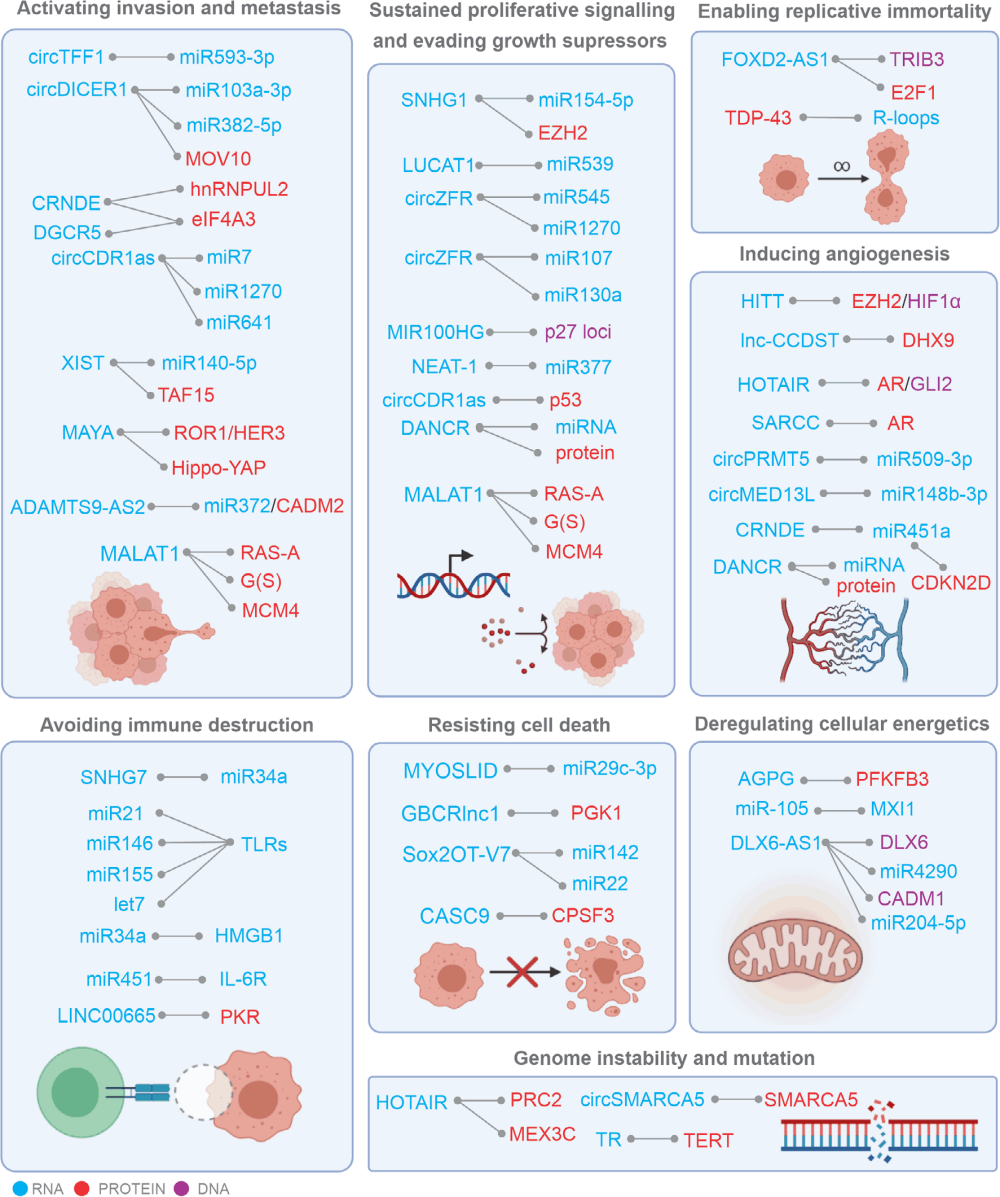
Specific examples of RNA interactions, discussed in the text, functioning within hallmarks of cancer including activating invasion and metastasis, sustained proliferative signaling, enabling replicative immortality, evading growth suppressors, inducing angiogenesis, avoiding immune destruction, resisting cell death, deregulating cellular energetics, and genome instability and mutations. Interacting partners are shown in blue (RNA), red (protein), and purple (DNA). Created with BioRender.com.

Next generation sequencing has revealed that a significant portion of the mutations associated with cancer development lie within the non‐coding region of the human genome. Mutation that has a particular effect on the expression of lncRNAs may, in turn, regulate various cancer phenotypes by interacting with DNA, RNA, and proteins (Grixti & Ayers, 2020; Schmitt & Chang, 2016). The best‐studied RNA–RNA interaction type is that of microRNA (miRNA) and messenger RNA (mRNA). The miRNAs are involved in the regulation of a variety of biological processes, such as cell cycle, differentiation, proliferation, apoptosis, stress tolerance, energy metabolism, immune response, oncogenesis, and drug resistance (Si et al., 2019), therefore affecting all hallmarks of cancer. Additionally, secretion of miRNAs can impact distant cell signaling or promote the formation of a niche that sustains a distant tumor microenvironment (Alečković & Kang, 2015). Deregulated microRNA expression favors acquisition of cancer hallmark traits as well as transforming the tumor microenvironment, leading to tumor development and progression (Manasa & Kannan, 2017). While some miRNAs and non‐coding RNAs have been documented to effect a single hallmark of cancer, there are examples, including the let‐7 miRNA family, which are involved in disruption of many processes/pathways/nodes (Figure 4; Feng & Tsao, 2016). For example, the decrease in let‐7 miRNAs, found in many cancers, results in overexpression of their oncogenic targets such as MYC, RAS, HMGA2, BLIMP1, among others (Balzeau et al., 2017).

Recently, a modified CLASH method was used to provide a comprehensive and unbiased snapshot of direct miRNA–RNA target interactions (Kozar et al., 2021). It was revealed that most miRNA–target interactions deviate from the central dogma of dependence on miRNA seed sites. Over 60% of these interactions occurred via non‐canonical seed pairing with a strong contribution of the 3′ miRNA sequence, and over 50% displayed a clear bias toward the coding sequence of mRNAs. This highlights the importance of noncanonical interactions, revealing further layers of complexity of post‐transcriptional gene regulation, thus expanding the pool of miRNA–target interactions, which have, so far, been omitted in the cancer field.

Many potential cancer‐related lncRNAs can act as scaffolds, interacting physically with other RNA species, resulting in a direct impact on cell signaling cascades (T. Zhao et al., 2015). Recently, lncRNA *bladder cancer‐associated transcript 1* (*BLACAT1*) has been observed to exert oncogenic effects on cancers, including glioma, breast cancer, lung cancer, hepatocellular carcinoma, gastric cancer, colorectal cancer, ovarian cancer, cervical cancer, and osteosarcoma (W. Han et al., 2020). It was suggested that *BLACAT1* can work through competitive binding of shared miRNAs, like *miR‐142‐5p* (D. Dai et al., 2019), *miR‐144* (Ye et al., 2017), *miR‐485‐5p* (Peng et al., 2019), *miR‐424*, and *miR‐143* (Cheng et al., 2020), thus affecting multiple genes, as well as being involved in the functions of the *miR‐605‐3p/VASP* (cells migration and proliferation; N. Liu, Hu, et al., 2019), *miR‐150‐5p/CCR2* (cells survival and motility; X. Hu et al., 2019), *miR‐17/ATG7* (cell death; F. Huang et al., 2018), *miR‐361/ABCB1* (multidrug resistance; X. Wu et al., 2018), and *miR‐519d‐3p/RPS15A* (angiogenesis; Yang et al., 2020) axes.

A recently discovered group of RNA molecules, called circular RNAs (circRNAs), have been implicated as miRNA sponges in a variety of cancers, including glioblastoma (Sun et al., 2020; J. Zhu et al., 2017) prostate cancer and others, as revealed by recent high‐profile publications (S. Chen, Huang, et al., 2019; Greene et al., 2019; Nigam et al., 2019). It was shown that the circRNA–miRNA–mRNA network is associated with the carcinogenesis of hepatocellular carcinoma, which might aid in the identification of molecular biomarkers and therapeutic targets (X. Lin & Chen, 2018). The network contained 60 circRNA–miRNA pairs and 4982 miRNA–mRNA pairs, including 29 circRNAs, 16 miRNAs, and 1249 mRNAs. Gene Ontology (GO) and Kyoto Encyclopedia of Genes and Genomes (KEGG) pathway analysis revealed the network might be involved in the procession of carcinogenesis such as affecting cell proliferation, cell cycle progression, and the P53 signaling pathway.

*Differentiation antagonizing non‐protein coding RNA* (*DANCR*) is an emerging therapeutic target in cancers. This lncRNA promotes the functions of vital components in the oncogene network by sponging their corresponding microRNAs or by interacting with various regulating proteins (S.‐J. Jin, Jin, et al., 2019). *DANCR* is a typical oncogenic lncRNA, overexpressed in various tumor cells. It can regulate the progression of numerous cancers by modulating the expression or activity of downstream targets and consequently promoting hallmarks of cancer including cell proliferation, cell motility, tumor angiogenesis, and cell viability. The vast majority of *DANCR*‐binding proteins, identified using the CARPID method, were associated with extracellular exosomes, denoting the presence of *DANCR* in this specialized cellular compartment and suggesting a potential for this interaction to impact remote sites in the body (W. Yi et al., 2020).

Another well‐studied lncRNA, *metastasis associated lung adenocarcinoma transcript 1* (*MALAT1*), is known to play either oncogenic or tumor‐suppressive roles in cancers (Q. Chen, Zhu, & Jin, 2020), inducing affecting cancer cell proliferation, migration, and invasion in vitro and tumor metastasis. A CARPID‐derived protein interactome study of *MALAT1* (W. Yi et al., 2020) identified guanine nucleotide‐binding protein G(S) protein, involved in cell proliferation and migration (X. Jin, Zhu, et al., 2019); Ras‐like proto‐oncogene A (RAS‐A); and minichromosome maintenance complex component 4 (MCM4) of the family of cancer‐contributing proteins (Y. Wang, Chen, et al., 2020).

These observations emphasize the complexity of networks targeted by conventional therapies elucidating additional master regulators and their dependency on cellular origin. Additionally, each of RNA can have few targets influencing functions of cancer trigger proteins either directly or indirectly, through several layers of interaction. To gain a deeper understanding of the role of RNA interactions in cancer we took a closer look at cancer hallmarks‐specific interactomic events (Figures 1b and 4).

### 4.2 Sustained proliferative signaling and evading growth suppressors

Cancer cells, unlike normal cells, can proliferate in the absence of stimulatory growth signals, through generation of many of their own growth signals, inducing autocrine hormone signaling. In addition, cancer cells can resist paracrine anti‐growth signals acquiring unregulated proliferative capacity.

*Small nucleolar RNA host gene 1* (*SNHG1*) acted as a sponge for *miR‐154‐5p*, reducing its ability to repress cell cycle regulator cyclin D2 (*CCND2*) expression in colorectal cancer (M. Xu, Chen, et al., 2018). Additionally, *SNHG1* interacts with EZH2 component of Polycomb Repressive Complex 2 (PRC2) and can thus modulate the histone methylation of promoter of Kruppel‐like factor 2 (*KLF2*) and Cyclin dependent kinase inhibitor 2B (*CDKN2B*) in the nucleus promoting colorectal cancer cell growth by affecting cell cycle progression and apoptosis.

*A circular RNA from the ZFR gene* (*CircZFR*) inhibited gastric cancer (GC) cell propagation, cell cycle, and promoted apoptosis by sponging *miR‐107*/*miR‐130a*, involved in cell propagation and impeded apoptosis through targeting *PTEN* (T. Liu et al., 2018). PTEN is the most important negative regulator of the PI3K signaling pathway and can regulate proliferation and cell survival. It also plays an intriguing role in regulating genomic stability, cell migration, stem cell self‐renewal, and the tumor microenvironment (Milella et al., 2015; Molinari & Frattini, 2014). In another report, *circZFR* directly interacts with *miR‐545* and *miR‐1270*, leading to upregulation of WNT5A (Wnt Family Member 5A) and promoting the progression of bladder cancer (L. Luo et al., 2020). These reports emphasizes the context‐dependent impact of these interactions and influence of additional unknown modulators.

It was shown that lncRNA *Mir‐100‐Let‐7a‐2‐Mir‐125b‐1 Cluster Host Gene* (*MIR100HG*) promotes cell proliferation in triple‐negative breast cancer through triplex formation with *P27* loci (S. Wang et al., 2018). The P27 (cyclin‐dependent kinase inhibitor 1B) inhibits cyclin/cyclin‐dependent kinase (CDK) complexes and halts cell cycle progression and regulates invasion and migration of cancer cells, functioning as either an oncoprotein or a tumor suppressor (Currier et al., 2019). Knockdown of *MIR100HG* decreased cell proliferation and induced cell arrest in the G1 phase, whereas overexpression of *MIR100HG* significantly increased cell proliferation.

*TP53* (tumor protein P53) is a critical tumor suppressor activated in response to many stress stimuli, including activation of oncogenes and DNA damage. Upon activation, P53 directly regulates the transcription of ~500 genes, indirectly regulates many additional genes and thereby controls diverse cellular processes, induces apoptosis in non‐transformed cells mostly by direct transcriptional activation of the pro‐apoptotic protein p53 upregulated modulator of apoptosis (PUMA) and, to a lesser extent, phorbol‐12‐myristate‐13‐acetate‐induced protein 1 (PMAIP1; Aubrey et al., 2018). CircRNA *CDR1as* functions as a tumor suppressor through binding directly to P53 at its DNA‐binding domain to restrict main negative regulator of P53 (MDM2) interaction (Lou et al., 2020). Thus, *CDR1as* binding disrupts the P53/MDM2 complex to prevent P53 from ubiquitination and degradation, leading to tumor growth inhibition. However, it has little impact in cells where P53 is absent or mutated, which includes 50%–60% of cancers (Baugh et al., 2018).

The long non‐coding RNA *nuclear enriched abundant transcript* (*NEAT‐1*) regulates *E2F3* expression by competitively binding to *miR‐377* in non‐small cell lung cancer and its expression is increased in multiple cancers (J. Zhang et al., 2017). *miR‐377* was shown to target *FZD4*, a gene important for epithelial‐to‐mesenchymal transition, to affect migration and invasion, and to target cell cycle kinase (*CDK6*) and E2F transcription factor 3 (*E2F3*), a potent transcriptional inducer of cell‐cycle progression (Zehavi et al., 2015).

### 4.3 Resisting cell death

Apoptosis can be induced in cancer cells through intrinsic and extrinsic pathways, which converge on the regulation of caspase‐dependent proteolysis of thousands of cellular proteins, membrane blebbing, and endonucleolytic cleavage of chromosomal DNA (Carneiro & El‐Deiry, 2020). Anti‐apoptotic proteins including BCL‐2 (B‐cell lymphoma 2), MCL‐1 (myeloid cell leukemia‐1), BCL‐W, BCL‐XL, and BCL2A1 promote cell survival (Shahverdi et al., 2020). The lncRNA *MYOSLID* functions as a competing endogenous RNA to regulate *MCL‐1* expression by sponging *miR‐29c‐3p* in gastric cancer (Y. Han et al., 2019).

In cancer cells, autophagy suppresses tumorigenesis by inhibiting cancer‐cell survival and inducing cell death, but it can also facilitate tumorigenesis by promoting cancer‐cell proliferation and tumor growth (Yun & Lee, 2018). Metabolism of the anticancer drug doxorubicin (Dox) leads to release of reactive oxygen species that can lead to lipid peroxidation and membrane damage, DNA damage, oxidative stress, and triggers apoptotic pathways of cell death (Thorn et al., 2011).

The lncRNA *GBCDRlnc1* interacts with phosphoglycerate kinase 1 (PGK1) and inhibits its ubiquitination in Dox‐resistant gallbladder cancer cells, affecting autophagy and chemoresistance of gallbladder cancer cells (Cai et al., 2019). Another lncRNA, *Sox2OT‐V7*, was shown to promote doxorubicin‐induced autophagy and chemoresistance in osteosarcoma via tumor‐suppressive *miR‐142/miR‐22*. The *Sox2OT‐V7/miR‐142/miR‐22* axis modulates autophagy in osteosarcoma cells by regulating *ULK1* (Unc‐51 like autophagy activating kinase 1), *ATG4A* (autophagy‐related 4A cysteine peptidase), and *ATG5* (autophagy‐related 5) genes (K. Zhu, Yuan, et al., 2020).

CPSF3 (Polyadenylation specificity factor 3) protein interacts with a lncRNA *CASC9* (Cancer Susceptibility 9), and knockdown of *CPSF3* mimicked the effects of *CASC9* knockdown in colorectal cancer cells. Furthermore, *CASC9* exerts its oncogenic activity by modulating *TGFβ2* (transforming growth factor beta‐2) mRNA stability and upregulating the levels of TGFβ2 and TERT (telomerase reverse transcriptase), resulting in an increase in phosphorylated SMAD3 (*SMAD* family member 3), activation of TGF‐β signaling, and enhanced TERT complex function in colorectal cancer cells (K. Luo et al., 2019). Inhibition of *CPSF3* upregulates apoptosis and causes tumor‐selective stasis, it prevents the release of newly synthesized pre‐mRNAs, resulting in read‐through transcription and the formation of DNA–RNA hybrid R‐loop structures (Ross et al., 2020). R‐loops are three‐stranded (triplex) nucleic acid structures, composed of an RNA–DNA hybrid and a non‐template, single‐stranded DNA. They are involved in regulating gene expression, class‐switch recombination, telomere stability, and mitochondrial DNA replication (J. P. Wells et al., 2019). Unscheduled R‐loop accumulation can drive DNA replication stress and genome instability. The CPSF complex is composed of at least five proteins: CPSF1, CPSF2, CPSF3, CPSF4, and FIP1L1 (factor interacting with PAPOLA and CPSF1) that recognizes the canonical nucleotide sequence AAUAAA in a newly synthesized pre‐messenger RNA (pre‐mRNA). Pre‐mRNA is cleaved by CPSF3, the catalytic component of the complex also known as CPSF73.

### 4.4 Enabling replicative immortality

Complete and accurate DNA replication is fundamental to cellular proliferation and genome stability and the obstacles that delay, prevent, or terminate DNA replication cause DNA replication stress (Ubhi & Brown, 2019). Loss of TDP‐43 (TAR DNA‐binding protein 43) increases DNA replication stress and damage, compromises cell viability, via increase of R‐loop formation in a transcription‐dependent manner (Wood et al., 2020). TDP‐43 nucleic‐acid‐binding and self‐assembly activities are important in inhibiting R‐loop accumulation and preserving normal DNA replication. It was suggested that TDP‐43‐regulates miRNAs and may play multifaceted roles in the pathogenesis of cancer (X. Chen et al., 2018).

The lncRNA *LUCAT1* (lung cancer‐associated transcript 1) acts as a molecular sponge of *miR‐539* in pancreatic ductal adenocarcinoma increasing cell proliferation, cell cycle progression, and invasion (Nai et al., 2020). In bladder cancer, lncRNA *FOXD2‐AS1* (*FOXD2* adjacent opposite strand RNA 1) forms an RNA–DNA complex with the promoter of *TRIB3* (Tribbles Pseudokinase 3), that leads to the activation of AKT (Serine–Threonine Protein Kinase), which further increases the expression of *E2F1* (E2F transcription factor 1), a vital transcription factor involved in the G/S transition (F. Su et al., 2018). The AKT serine/threonine kinase, also known as protein kinase B (PKB), is an oncogenic protein that regulates cell survival, proliferation, growth, apoptosis, and glycogen metabolism (Song et al., 2019). Interestingly, E2F1 could bind to the *FOXD2‐AS1* promoter region and subsequently enhance its transcriptional activity, indicating that *FOXD2‐AS1*/AKT/E2F1 form a feedback loop. TRIB3 promotes chronic inflammation and cancer by interacting with intracellular signaling and functional proteins. The interaction of TRIB3 and the autophagic receptor P62 (Sequestosome 1) interferes with the degradation of autophagy and the ubiquitin–proteasome system to control the initiation and progression of cancer.

### 4.5 Inducing angiogenesis

Due to their high metabolic rate cancer cells require lots of nutrients. To achieve this, tumor vascularization occurs through several distinct biological processes, orchestrated by a range of secreted factors and signaling pathways and can involve participation of non‐endothelial cells, such as progenitors or cancer stem cells (Lugano et al., 2020).

Recently, a long non‐coding RNA named *HITT* (HIF‐1α inhibitor at translation levels) was identified to play roles in modulating hypoxia‐mediated angiogenesis and tumor growth in vivo (X. Wang, Wang, et al., 2020). Mechanistic studies revealed that *HITT* inhibits *HIF‐1α* (hypoxia‐inducible factor 1 alpha) transcription by guiding EZH2 (enhancer of Zeste 2 polycomb repressive complex 2 subunit) through the formation of an RNA–DNA triplex with the *HIF‐1α* promoter.

A long noncoding RNA, named *CCDSTlnc* (cervical cancer DExH‐box helicase 9 suppressive transcript), is significantly downregulated in cervical cancer tissues, and binds to upregulated pro‐oncogenic DHX9 (DExH‐Box Helicase 9) protein, promoter of motility, and angiogenesis (X. Ding et al., 2019). The *CCDSTlnc* and DHX9 interaction creates a scaffold to facilitate the formation of MDM2 (E3 ubiquitin ligase) and DHX9 complex, promoting degradation of the latter through the ubiquitin proteasome pathway.

VEGF‐A (vascular endothelial growth factor A) is a member of a family including VEGF‐B, VEGF‐C, and platelet‐derived growth factor (PDGF), playing an important role in vasculogenesis (Apte et al., 2019). It is well known PDGFA is a subtype of PDGF, which is an oncogene that can regulate a variety of cell processes by activating their cognate receptors, including cell proliferation, apoptosis, migration, invasion, angiogenesis, and metastasis, which are involved in the progression of human tumors (X. Ding et al., 2019). The lncRNA *HOTAIR* interacts with AR (androgen receptor) and they cooperatively bind to *GLI2* promoter, increasing its transcription activity in renal cell carcinoma cells, enhancing the expression of *GLI2* downstream genes, such as *VEGFA*, *PDGFA*, and cancer stem cell transcription factors, promoting tumor angiogenesis and cancer stemness both in vitro and in tumor xenografts (Bai et al., 2021). On the other hand, lncRNA *SARCC* was found to bind and destabilize AR protein in renal cell carcinoma, leading to reversing the effect of transcriptional repression by *miR‐143‐3p*, inhibiting its downstream signals such as AKT, MMP‐13 (matrix metalloproteinase 13), K‐RAS (Kirsten rat sarcoma virus) and P‐ERK (PKR‐like endoplasmic reticulum kinase; Zhai et al., 2017).

CircRNA *PRMT5* (protein arginine methyltransferase 5) is upregulated in breast cancer tissues and cells and increases *TCF7L2* (transcription factor 7 like 2) expression by acting as a *miR‐509‐3p* sponge (D. Wu et al., 2021), in turn activating the phosphoinositide 3‐kinase (PI3K)/AKT pathway. Knockdown of *circPRMT5* suppresses cell proliferation and angiogenesis and increases cell apoptosis in breast cancer while *TCF7L2* overexpression reverses the antitumoral effects of *circPRMT5* knockdown on breast cancer cell processes. Similarly, *CircMED13L* (Mediator Complex Subunit 13L) was found to act as the sponge of *miR‐148b‐3p* in breast cancer cells, thus exerting the tumor‐suppressive effects (D. Wu et al., 2021, p. 13). The study showed that *miR‐148b‐3p* directly targeted *PTEN*, which negatively regulates PI3K/AKT pathway, thus promoting tumor growth in breast cancer (J. Yu, Sun, et al., 2019).

The lncRNA *CRNDE* (colorectal neoplasia differentially expressed), significantly upregulated in pancreatic cancer, promotes the progression and angiogenesis through sponging *miR‐451a* (H.‐Y. Zhu et al., 2021). It was shown that *miR‐451a* directly interacted with and negatively regulated *CDKN2D* (cyclin‐dependent kinase inhibitor 2D) expression. *CDKN2D* is a negative regulator of the cell cycle. *CDKN2D* protein, like other inhibitors of cyclin‐dependent kinases (INK4s), inhibits CDK4 (cyclin‐dependent kinase 4)–cyclin D1 activity in vivo, and induces G_1_ phase arrest (Felisiak‐Golabek et al., 2013).

### 4.6 Activating invasion and metastasis

Autophagy is a highly conserved self‐degradative process that has a key role in cellular stress responses and survival and has been shown to be involved in modulating tumor cell motility and invasion, cancer stem cell viability and differentiation, epithelial‐to‐mesenchymal transition, tumor cell dormancy, and escape from immune surveillance, with emerging functions in establishing the pre‐metastatic niche and other aspects of metastasis (Mowers et al., 2017).

Cancer‐derived extracellular vesicles (EVs), containing *miR‐181c* promote the destruction of blood–brain barrier through the abnormal localization of actin via the downregulation of its target gene, *PDPK1* (3‐phosphoinositide‐dependent protein kinase 1; Tominaga et al., 2015)*. PDPK1* degradation by *miR‐181c* leads to the downregulation of phosphorylated cofilin and the resultant activated cofilin‐induced modulation of actin dynamics. Taken together, these results showed a novel mechanism of cancer metastasis mediated by EVs. PDPK1 is known to phosphorylate AKT. However, recent studies have also shown that PDPK1 can activate many other members of AGC kinase family such as p70S6K (ribosomal protein S6 kinase beta‐1), SGK (serum/glucocorticoid regulated kinase), p90RSK (ribosomal protein S6 kinase), and the members of PKC (protein kinase C) family, independent of AKT (Nalairndran et al., 2020).

Knockdown of another circRNA, *circTFF1* (Trefoil Factor 1), overexpressed in breast cancer, hampered cell viability, colony formation, migration, invasion, cell cycle progression and enhanced cell apoptosis in vitro, and weakened tumor growth in vivo (Xie et al., 2020). It was shown that *circTFF1* functioned as a molecular sponge of *miR‐593‐3p*, which targets *FGFR3*. The fibroblast growth factor receptors (FGFR) comprise a family of transmembrane tyrosine kinase receptors that play vital roles in cell differentiation, growth, and angiogenesis through binding of their respective ligands, contributing to its carcinogenesis through signal transducer and activator of transcription protein (STAT), phosphatidylinositide 3‐kinases/protein kinase B (PI3K/AKT), and RAS/mitogen‐activated protein kinase (RAS/MAPK) pathways (L. Li et al., 2019). STAT proteins are a class of transcription factors that are activated by cytokines, growth factors, and other peptide ligands in response to diverse cytokine signals in the cytoplasm. Following activation, the STAT proteins translocate to the nucleus, binding their specific targets and serving as transcription factors (Gu et al., 2020). The RAS family of small GTPases comprises the three proteins in humans, HRAS, NRAS, and KRAS, converted from an inactive GDP‐bound state to an active GTP‐bound state by RAS guanine nucleotide exchange factors (GEFs) recruited to the plasma membrane, for example, upon activation of receptor tyrosine kinases. GTP‐bound RAS adopts a new conformation that binds a host of proteins (known as effectors) through their RAS binding domain (RBD), the most studied being RAF (Raf‐1 Proto‐Oncogene, Serine/Threonine Kinase) kinases of the MAPK (a mitogen‐activated protein kinase) pathway, the p110 subunit of PI3K and RalGEFs (Ral‐selective Guanine Nucleotide Exchange Factors), which transmit a series of proproliferative and other signals to the cell (S. Li et al., 2018).

It was found that *circDICER1* (*Dicer 1*, Ribonuclease III) acts as a molecular sponge to bind *miR‐103a‐3p/miR‐382‐5p* and impair their negative regulation of *ZIC4* in glioma‐exposed endothelial cells (Q. He et al., 2019). Furthermore, ZIC4 up‐regulates the expression of its downstream target *HSP90β* (heat shock protein 90 alpha family class B), and *HSP90* which promotes the cell viability, migration, and tube formation by activating PI3K/AKT signaling pathway. Additionally, RNA binding protein MOV10 (*Mov10* RISC complex RNA helicase) binds *circDICER1* and also regulates cells' viability, migration, and tube formation.

*CRNDE* and *DGCR5* (DiGeorge Syndrome Critical Region *Gene* 5) lncRNAs, highly expressed in EGFR‐TKI‐resistant lung cancer cells, bind eukaryotic translation initiation factor 4A3 (EIF4A3) in an overlapping manner (Takahashi et al., 2021). The *CRNDE* lncRNA downregulates the expression of *EIF4A3*, mucin 1 (*MUC1*), and phospho‐*EGFR* (Epidermal Growth Factor Receptor), reducing apoptotic activity. Moreover, *CRNDE* was reported to competitive bind nine different miRNAs, and was found able to form a functional complex with heterogeneous nuclear ribonucleoprotein U‐like 2 protein (hnRNPUL2) and direct the transport of hnRNPUL2 between the nucleus and cytoplasm, affecting not only proliferation, invasion and metastasis but also other hallmarks of cancer (Y. Lu et al., 2020).

Additionally, *CDR1as* contains over 70 *miR‐7* binding sites and can regulate gene activity by sponging *miR‐7* and promoting the proliferation and metastasis of cancer cells (Hansen et al., 2011; C. Jiang et al., 2020; Memczak et al., 2013; B. Xu, Yang, et al., 2018). *CDR1as* can sponge *miR‐1270* and upregulate the expression of *AFP* to promote tumor growth, invasion and metastasis in hepatocellular carcinoma (Y. Su et al., 2019) or *miR‐641* in cholangiocarcinoma (D. Li et al., 2020). It implies that *CDR1as* has different effects on cell growth in different cancer types and depending on expression levels.

*XIST* is known to contribute to progression of cervical cancer via regulating *miR‐140‐5p* (X. Chen, Xiong, et al., 2019). *miRNA‐140‐5p* has been demonstrated to play an important role in the initiation and progression of cancer, can inhibit tumor formation and metastasis of colorectal cancer and regulate the stem cells by suppressing the *WNT*, *SOX2*, and *SOX9* (SRY‐Box Transcription Factor 2 and 9) stem cell regulator pathways in breast cancer (D. Wu, Zhang, et al., 2019). Therefore, the result of *XIST‐miR‐140‐5p* is context‐dependent. In addition to known *XIST* interactors, CARPID also identified multiple new binding factors, including a transcription‐initiation factor TFIID subunit called TATA‐box binding protein associated factor 15 (TAF15; W. Yi et al., 2020). SOX2 is a cell‐fate determining transcription factor that has been functionally implicated in the induction and maintenance of pluripotent induced pluripotent stem (iPS) and embryonic stem (ES) cells, multipotent lineage‐committed progenitors, and tissue stem cells of mostly epithelial or neural fate. Moreover, SOX2 has been recognized as a powerful oncogene in various cancer types, where it regulates cancer stem cells (CSCs) and functionally relates to several other hallmarks. SOX2 is a powerful oncogene that functionally relates to cancer stemness and various further hallmark functionalities amongst which clonogenicity, tumorigenicity, endothelial‐to‐mesenchymal transition (EMT), cancer cell mobility, tumor cell dissemination, metastasis, chemotherapy resistance, and relapse (Schaefer & Lengerke, 2020).

It was shown that crosstalk between ROR1‐HER3 (Receptor Tyrosine Kinase Like Orphan Receptor 1, Erb‐B2 Receptor Tyrosine Kinase 3) and the Hippo‐YAP (Serine/Threonine Kinase 4, Yes‐associated protein) protein pathway promotes breast cancer bone metastasis and is dependent on binding lncRNA *MAYA* (C. Li et al., 2017). The receptor tyrosine kinase‐like orphan receptor 1 (ROR1) is an oncofetal glycoprotein involved in differentiation, proliferation, migration, and survival during the intrauterine development (Schiavone et al., 2020). The human EGFR family consists of four tyrosine kinase receptors (EGFR/HER1, HER2, HER3, and HER4) that stimulate growth signaling pathways involved in cell proliferation, growth, survival, and differentiation (Ogden et al., 2020). The hippo pathway possesses the unique capacity to lead to tumorigenesis and mutations and altered expression of its core components (MST1/2, LATS1/2, YAP, and TAZ) promote the migration, invasion, and malignancy of cancer cells (Y. Han, 2019).

The constructed novel competitive endogenous RNA (ceRNA) network and the potential regulatory axes might provide a novel approach for exploring the potential mechanisms of development in gastric cancer (Pan et al., 2019). The study, combining computational and experimental methods, showed that lncRNA *ADAMTS9‐AS2/miR‐372/CADM2* could act as a promising target for gastric cancer treatment. qRT‐PCR showed that *ADAMTS9‐AS2* (ADAM Metallopeptidase With Thrombospondin Type 1 Motif 9) knockdown remarkably increased *miR‐372* expression but reduced *CADM2* expression, whereas *ADAMTS9‐AS2* overexpression had the opposite effects. CADM2 (Cell Adhesion Molecule 2) is involved in cancer cell migration, invasion, and metastasis (L. Dai et al., 2020). CADM immunoglobulin super family is involved in the maintenance of cell adhesion, polarity, and tumor suppression (N. Liu, Yang, et al., 2019).

### 4.7 Deregulated cellular energetics

Transformed cells adapt metabolism to support tumor initiation and progression and changes in cell metabolism can contribute to transformation and tumor progression. Specific metabolic activities, (that are transforming, enabling, or neutral) can participate directly in the process of transformation or support the biological processes that enable tumor growth (Vander Heiden & DeBerardinis, 2017). The common feature of this altered metabolism is increased glucose uptake and fermentation of glucose to lactate known as the Warburg Effect (Liberti & Locasale, 2016). It is well‐recognized that lncRNAs and circRNAs regulate energy metabolism in cancer (Tan et al., 2021; T. Yu, Wang, et al., 2019).

The lncRNA *AGPG* (Actin Gamma 1 Pseudogene) is required for increased glycolysis activity and cell proliferation in esophageal squamous cell carcinoma (J. Liu et al., 2020). *AGPG* binds to and stabilizes 6‐phosphofructo‐2‐kinase/fructose‐2,6‐biphosphatase 3 (PFKFB3), protecting it from proteasomal degradation, leading to the accumulation of PFKFB3 in cancer cells, which subsequently activates glycolytic flux and promotes cell cycle progression.

Extracellular miRNAs have been recently implicated in the intercellular crosstalk (Tominaga et al., 2015). Breast‐cancer‐secreted, extracellular‐vesicle‐encapsulated miR‐105, which is induced by the oncoprotein MYC in cancer cells and in turn activates MYC signaling, through targeting *MXI1* (MAX interactor 1) gene in cancer‐associated fibroblasts (CAFs) to induce a metabolic program (W. Yan et al., 2018). The MYC proto‐oncogene functions as a transcription factor that coordinates many biological processes and its activation contributes to autonomous proliferation and growth, relentless DNA replication, increased protein biogenesis, global changes in cellular metabolism, activation of the angiogenic switch, suppression of the response to autocrine and paracrine regulatory programs, and a restraint of host immune responses (Gabay et al., 2014). MXI1 can antagonize the transcriptional activity of *MYC* by competing with MAX (MYC Associated Factor X) and plays essential roles in multiple biological processes which include cell growth and differentiation, cell cycle regulation, cell apoptosis, and radiosensitivity (Y. Huang et al., 2021).

Extensive involvement of multiple circRNAs in cancer metabolism was recently reviewed by Yu, Sun, et al. (2019). For example, it was shown that silencing of *circHIPK3*, which is abundant in pancreatic islets, decreased expression of *SLC2A2* (Solute Carrier Family 2 Member 2) that encodes GLUT2, one of the glucose transporters (GLUT) in charge of the import of glucide into the cells (Stoll et al., 2018). *CircHIPK3* can sponge many miRNAs, including *miR‐124*, which was shown to repress the expression of several enzymes and transporters of glycolysis in non‐small cell lung cancer cells (X. Zhao et al., 2017). C‐MYC, together with HIF‐1 can elevate the level of the glycolytic components, like GLUT1, HK2 (Hexokinase 2), and PDK1 (Phosphoinositide‐Dependent Protein Kinase 1; Z. Li & Zhang, 2016). Expression of C‐MYC is regulated by *miR‐145* (F. Wang et al., 2013) and *miR‐34a*, that can be sponged by *circBIRC6* (Baculoviral IAP Repeat Containing 6; C.‐Y. Yu et al., 2017).

It was shown that lncRNA *DLX6‐AS1* (DLX6 Antisense RNA 1) formed a DNA–RNA triplex structure with the promoter of the *DLX6* transcription factor via recruitment of P300/E2F1 (E1A Binding Protein P300) acetyltransferase in endometrial cancer cells (H. Zhao & Xu, 2020). Cancer cells growth in vivo can be suppressed by silencing either *DLX6‐AS1* or *DLX6*. It was also shown that *DLX6‐AS1* regulates tumor growth and aerobic glycolysis in gastric cancer by targeting *miR‐4290* and *PDK1* (Qian et al., 2021). It was also shown that down‐regulation of *DLX6‐AS1* may inhibit the stem cell properties of liver cancer stem cells through upregulation of *CADM1* by suppressing the methylation of the *CADM1* promoter and inactivation of the STAT3 signaling pathway (D.‐M. Wu, Zheng, et al., 2019). In other studies, downregulation of *DLX6‐AS1* inhibited gastric cancer cell proliferation, migration, invasion, and EMT in vitro, through competing endogenous RNA (ceRNA) by binding *miR‐204‐5p* and upregulating *OCT1* (Solute Carrier Family 22 Member 1). Moreover, the transcription factor *OCT1* was confirmed to enhance *DLX6‐AS1* expression by targeting the promoter region (Liang, Li, et al., 2020).

### 4.8 Avoiding immune destruction

Tumor‐mediated escape mechanisms include alterations in (1) the generation, processing, and presentation of T cell epitopes derived from tumor‐associated antigens by human leukocyte antigen (HLA) class I and/or class II molecules, (2) signal transduction pathways, (3) the expression of costimulatory and co‐inhibitory molecules, (4) the expression of apoptosis‐related molecules, and (5) the secretion of immune‐suppressive mediators as well as highly specialized double‐membrane vesicles called exosomes, which play a crucial role in conditioning the local microenvironment and intercellular communication (Eichmüller et al., 2017).

*SNHG7* (Small Nucleolar RNA Host *Gene* 7) served as a competing endogenous RNA of *miR‐34a*, and *SIRT1* (Sirtuin 1) was identified as a direct target of *miR‐34a*. Cell pyroptosis was evaluated by TUNEL (Terminal Deoxynucleotidyl Transferase dUTP Nick End Labeling) and lactate dehydrogenase release assays. *SNHG7* knockdown reduced *SIRT1* expression, but increased the expression levels of *NLRP3* (NLR family pyrin domain containing 3), caspase‐1, and interleukin‐1β, leading to pyroptosis. NLRP3 also called the inflammasome, is a multimolecular protein complex that performs a specific function for the cytoplasm in the host's innate immunity by secreting inflammatory factors and promoting maturation, such as for interleukin‐1β (IL‐1β) and interleukin‐18 (IL‐18; Y. He et al., 2016). *SNHG7* knockdown‐induced effects were enhanced by *miR‐34a* upregulation. In summary, the present study indicated that the *SNHG7/miR‐34a/SIRT1* axis contributed to NLRP3‐dependent pyroptosis during liver cancer (Z. Chen, He, et al., 2020). Pyroptosis is characterized by caspase 1‐dependent formation of plasma‐membrane pores, which leads to pathological ion fluxes that ultimately result in cellular lysis and release of inflammatory intracellular contents (Bergsbaken et al., 2009). Pyroptosis, or caspase 1‐dependent cell death, is inherently inflammatory, is triggered by various pathological stimuli, such as stroke, heart attack, or cancer, and is crucial for controlling microbial infections.

Toll‐like receptors (TLRs) are evolutionarily conserved molecules that initiate the signaling cascade of immune response against a wide variety of pathogens and are also expressed in tumor cells and their microenvironments (Cen et al., 2018). An increasing number of studies have demonstrated that several miRNAs, including *miR‐21*, *miR‐146*, *miR‐155*, and *let‐7* family, target TLRs, or proteins in TLR signaling pathways that are involved in the regulation of various processes, such as inflammation, T‐cell activation, cellular infiltration, and immunity development (Bayraktar et al., 2019).

HMGB1 (High Mobility Group Box 1) is an oncogene that induces inflammation and facilitates tumorigenesis and metastasis and its downregulation by *miR‐34a* is sufficient to suppress proliferation, migration, and invasion of human cervical and colorectal cancer cells (Chandrasekaran et al., 2016). Another miRNA, *miR‐451*, has an essential function in cell growth and differentiation. Down‐regulation of *miR‐451* increased the expression of its target gene, *IL‐6R* (an inflammatory cytokine) in both RKO and HeLa cells. It was discovered that *miR‐451* has a tumor suppressor activity by targeting *IL‐6R*, so that its down‐regulation can induce inflammation, invasion, angiogenesis, and proliferation in cervical cancer cells (Sadri Nahand et al., 2020).

Innate immunity and inflammation often promote tumorigenesis and malignant progression of nascent cancer and inflammation‐related biological processes influence all stages of cancer development and treatment (Hou et al., 2021). The nuclear factor kappa B (NF‐κB) signaling pathway is important for linking inflammation and tumorigenesis. The lncRNA *LINC00665* physically interacts with the dsRNA‐activated protein kinase (PKR), enhances its activation, and maintains its protein stability by blocking ubiquitin/proteasome‐dependent degradation, resulting in a positive feedback regulation of NF‐κB signaling in hepatocellular carcinoma cells (J. Ding et al., 2020).

### 4.9 Genome instability and mutation

Loss of DNA repair genes leads to genome instability, defined as abnormally high rates of mutation. This, in, turn increases mutation rates at other genomic sites, leading to cellular transformation (Tubbs & Nussenzweig, 2017). The telomerase holoenzyme includes a unique reverse transcriptase TERT, an essential RNA (TR), and several species‐specific proteins required for proper function in vivo (Q. Zhang et al., 2011). Interaction between *TR* (Telomerase RNA) and TERT complex underlies the process of correct telomers synthesis, in the moment of synthesis creating protein‐RNA–DNA construct. The shortening of telomeres can exert a tumor‐suppressive effect through the proliferation arrest induced by activating the kinases ATM (*ATM* Serine/Threonine *Kinase*) and ATR (*ATR* Serine/Threonine *Kinase*) at unprotected chromosome ends, while loss of telomere protection can lead to telomere crisis, which is a state of extensive genome instability that can promote cancer progression (Maciejowski & de Lange, 2017).

In HBV (Hepatitis B Virus) replicating cells, including virus‐associated hepatocellular carcinoma, a core subunit of Polycomb repressive complex2 (PRC2), undergoes proteasomal degradation. This process requires the long noncoding RNA, Hox transcript antisense intergenic RNA (*HOTAIR*). Intriguingly, *HOTAIR* interacts with PRC2 and also binds RNA‐binding E3 ligase (MEX3C), serving as a ubiquitination scaffold. It was identified that the RNA helicase, DEAD box protein 5 (DDX5), is a regulator of Polycomb stability and PRC2‐mediated gene repression, acting by regulating RNA–protein complexes formed with *HOTAIR* (H. Zhang et al., 2016).

It was shown recently that the ATP‐dependent chromatin remodeling INO80 (*INO80* Complex ATPase Subunit) complex promotes resolution of R‐loops to prevent replication‐associated DNA damage in cancer cells, while depletion of INO80 in prostate cancer cells leads to increased R‐loops (Prendergast et al., 2020). Additionally, cancer survival time is associated with the mRNA expression of R‐loop binding proteins, and expression of R‐loop genes correlates with drug response in cancer (Boros‐Oláh et al., 2019). Therefore, R‐loop binding proteins are considered to offer promise as new markers and targets for cancer therapy. It was also demonstrated that *circSMARCA5* can bind to its parent gene locus, forming an R‐loop, which results in transcriptional pausing, resulting in the production of a truncated non‐functional protein (X. Xu et al., 2020). Additionally, the overexpression of *circSMARCA5* in the same study was shown to be sufficient to improve sensitivity to cytotoxic drugs. On the other hand, recent studies shown that tumor treating fields (TTFields), a non‐invasive physical modality of cancer therapy can induce mitotic aberrations and DNA damage/replication and increase R‐loop formation (Karanam et al., 2020). Damaging DNA–RNA interaction can therefore occur in cancer therapy and should be taken into consideration.

### 4.10 Pathogen–host interactions

Approximately 15%–20% of cancers are associated with viral infections, with seven widely‐accepted oncoviruses identified: Epstein–Barr Virus (EBV), Human Papillomavirus (HPV), Hepatitis B and C viruses (HBV and HCV), Human T‐cell lymphotropic virus‐1 (HTLV‐1), Kaposi's sarcoma‐associated herpesvirus (KSHV)/Human Herpesvirus‐8 (HHV‐8), and Merkel Cell Polyomavirus (MCPyV). As obligatory intracellular parasites, viruses encode RNAs and proteins that interact with and reprogram host cellular signaling pathways including proliferation, differentiation, cell death, genomic integrity, and recognition by the immune system (McLaughlin‐Drubin & Munger, 2008). Both coding RNAs, and non‐coding RNAs (miRNAs and circRNAs) originate from the viral genomes and have been implicated in various hallmarks of cancer. Specifically, herpes simplex virus 1 (HSV‐1) expresses numerous miRNAs and deregulates the expression of host mRNAs and miRNAs. Several HSV‐1 miRNAs are abundantly expressed in latency, some of which are encoded antisense to transcripts of important productive infection genes, indicating their roles in repressing the productive cycle and/or in maintenance/reactivation from latency (Cokarić Brdovčak et al., 2018). Infection of human cells with different oncoviruses (KSHV, EBV, and CMV) has been shown to induce host circular RNAs, including I_circ_0001400, which was shown to interact with host protein and RNA molecules and promote viral latency (Tagawa et al., 2023).

## 5 COMPUTATIONAL PERSPECTIVE

The complexity of studies described above demands specialized computational pipelines and platforms, ideally employing artificial intelligence and neural networks—a synthesis of disciplines in its infancy in this field. While greatly inspirational to a few specialists, systems concepts have remained largely ignored by most molecular biologists, at least until empirical observations could be gathered to validate them (Vidal et al., 2011). As predicted a few years ago, currently, experimental and computational methods increase the resolution of interaction studies using interaction networks to connect sequence and structural information, and to understand the biological consequences of disease‐associated mutations, which will hopefully lead to more effective therapeutic strategies CSCs (Ryan et al., 2013).

**FIGURE 5:**
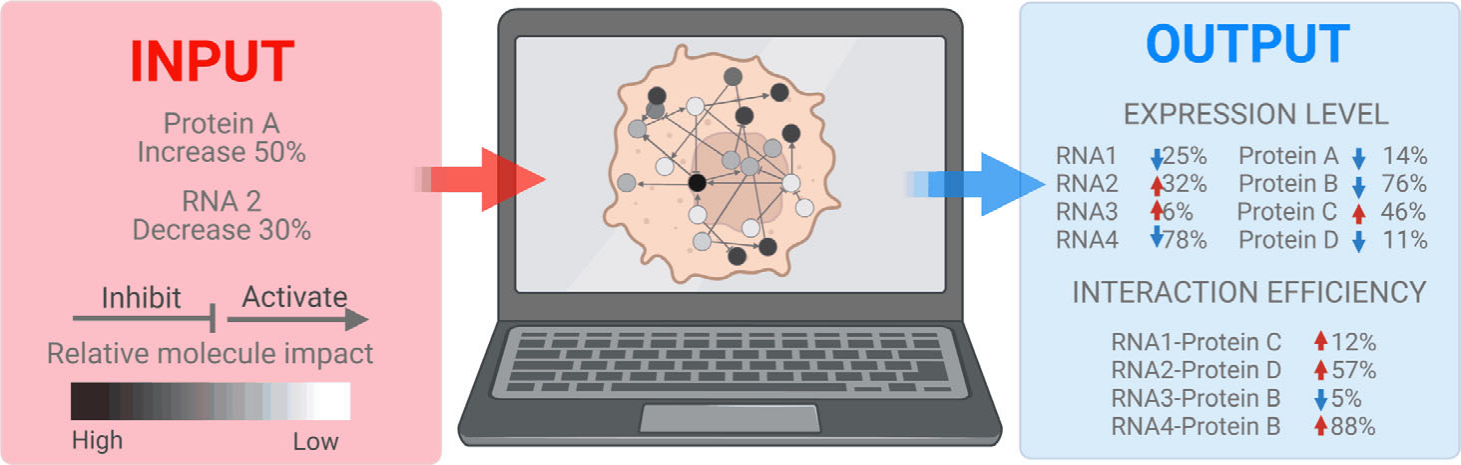
“Digital cell” model of hypothetical artificial intelligence software for incorporating all existing and future “omics” dataset for prediction of changes in cellular homeostasis driven by increase/decrease of expression of particular gene. Input information (on the left) stratifying and quantifying changes of expression on RNA or protein level, type of effect (inhibitory/stimulatory) and significance of interaction, understood as the control potential of the molecule and it's known involvement in many interactions. Suggested output (on the right) shows changes in interactions levels of particular hypothetical molecules. Created with BioRender.com.

Development of high‐throughput interactomic methods and computational data cohesion is essential for an evaluation of the exact roles of each molecule in the context of cellular unity. Cancer‐specific prediction method named NECARE, although limited to network‐based cancer protein–protein interaction predictions, maps the perturbation of cancer interactome (Qiu et al., 2021). Establishing a digital interactive cell model, incorporating all major interactomic cellular events, is a next step in cellular networks research (Figure 5). The problem with high‐throughput methods for interactome capture is that it is generally assumed that an interaction is true if it is represented by many unique hits. While it is hard to deny this logic, interactions that occur fast will have lower capture probability, thus resulting in a greater risk of static interactions being over‐represented, compared with functional, short‐lived interactions.

Interactomic studies are supported by increasing numbers of bioinformatics tools, critical to deconvolute functional networks. RNAInter establishes a repository of integrated experimentally validated and computationally predicted RNA‐associated interactions providing a comprehensive resource for researchers and paves the way to investigate the regulatory landscape of cellular RNAs (J. Kang et al., 2021; Y. Lin et al., 2020). Li et al., 2021 developed e‐MutPath (edgetic Mutation‐mediated Pathway Perturbations), a network‐based computational method to identify candidate “edgetic” mutations that perturb functional pathways and could be used to distinguish disease risk factors from neutral elements and to stratify disease subtypes with clinical relevance. Recently, Zhu et al. constructed an RNA–RNA interaction (RRI) network by extensively collecting reported RNA–RNA interactions and then comparing it with the protein–protein interaction and the gene co‐expression networks in breast cancer (X. Zhu, Liu, et al., 2020). They showed that the RNA–RNA interaction network could be anticipated as a complement to the well‐studied protein–protein interactions and co‐expression network methods.

## 6 INTERACTIONS AS THERAPEUTIC TARGETS

Considering the plethora of possible interactions involving RNA molecules, it is likely to look for future targeted therapies in this growing field on the edge of molecular and systems biology. Each interaction presents itself as a unique structural feature and, as such, could be recognized and disrupted or prevented. Therefore, future research approaches should focus on identifying pathogenic interactions in cancer, not pathogenic molecules. This approach could minimize side effects of chemotherapies if the target is localized only in cancer cells. Conceivably, engineering an approach to disrupt these pathological RNA interactions only in the target cells, that is, the cancer, can become a new, broad‐spectrum therapeutic strategy with reduced the side effects of the treatment. There are a number of strategies in pre‐clinical phase for disrupting RNA–RNA interactions, primarily focussed on mimics/antagonists of miRNAs and ncRNAs, which was well summarized by Liang, Zhang, et al. (2020). One specific example is using the inhibitor KH‐3 to disrupt the interaction of the RNA‐binding protein Hu antigen R (HuR) and *FOXQ1* (Forkhead Box Q1) mRNA, leading to inhibition of breast cancer invasion in high‐grade tumors with poor clinical outcome (X. Wu et al., 2020). Therefore, these RNA interactomic networks offer a plethora of unexplored potential targets for clinical intervention.

## 7 CONCLUSION

The sum‐total of all molecular interactions within and without the cell contribute to cellular homeostasis. These interactions depend on the structural properties, availability, and accessibility of the molecules and form the basis of a dynamic cellular equilibrium. A volatility, resulting from changes to the so‐called “molecular interactome,” can precipitate cellular‐level and organismal‐level perturbations, including disease. For many years, RNA molecules and their functional networks remained in the shadows of the functional proteome. Therefore, understanding the fundamentals underlying RNA molecular communications is a crucial task in the molecular biology of cancer. We are currently able to track and identify particular RNA interactions involved in all hallmarks of cancer. However, future efforts should be focused on global screening and functional analysis of healthy and pathogenic RNA interactions. Akin to chaos theory, disrupting the expression level of one molecule has consequences at many levels. Despite being a widely held belief, it is unlikely that in the crowded cellular environment each RNA or protein molecule will have a paucity of interacting partners and that these are stable. Consequently, not only the identification of interacting partners, but also the dynamism of such interactions—timing, competitors, localization, and stoichiometry—are important questions to address in cancer research.

Future studies should reveal intrinsic conformations of the RNAs at high resolution, and the ability of these RNAs, through their accessibility, to interact with partners (RNA, DNA, and protein) in trans. Once achieved, this framework can be exploited to model the potential consequences of somatic mutations, discriminate role in oncogenesis and help to understand reasons behind effective or non‐effective cancer treatments, including adding novel therapeutic targets. The expected technological advances will provide deep insight into the roles of RNA structures in human cells on a global scale, providing functional profiling of poorly explored interactomes.

## AUTHOR CONTRIBUTIONS

**Marta M. Gabryelska:** Conceptualization (lead); formal analysis (lead); funding acquisition (equal); investigation (lead); visualization (lead); writing – original draft (lead); writing – review and editing (equal). **Simon J. Conn:** Conceptualization (supporting); formal analysis (supporting); funding acquisition (equal); investigation (supporting); project administration (lead); supervision (lead); visualization (supporting); writing – original draft (supporting); writing – review and editing (equal).

## FUNDING INFORMATION

This research was funded by National Health and Medical Research Council (NHMRC) project grant funding schemes to S.J.C. (GNT1089167, GNT1144250), a Ray and Shirl Norman Cancer Research Trust grant awarded to S.J.C., Flinders Foundation Health Seed grant awarded to S.J.C. and FHMRI Kickstart ECR grant awarded to M.M.G. Fellowship support for S.J.C. was provided by the Australian Research Council Future Fellowship (FT160100318) and the NHMRC Investigator Leadership Grant (GNT1198014).

## CONFLICT OF INTEREST STATEMENT

The authors have declared no conflicts of interest for this article.

## RELATED WIREs ARTICLES

Computational biology of RNA interactions

Emerging roles of RNA‐RNA interactions in transcriptional regulation

Detecting RNA‐RNA interactome

## Data Availability

Data sharing is not applicable to this article as no new data were created or analyzed in this study.
